# Mixture design as a tool for improving full-to-empty particle ratios across various GOIs in rAAV production

**DOI:** 10.1038/s41434-025-00546-5

**Published:** 2025-06-20

**Authors:** Konstantina Tzimou, Pol Hulsbus-Andreu, Ece Bahar Yildirim, Lars K. Nielsen, Jesús Lavado-García

**Affiliations:** 1 The Novo Nordisk Foundation Center for Biosustainability, Technical University of Denmark, Lyngby, Denmark; 2 DTU Bioengineering, Technical University of Denmark, Lyngby, Denmark; 3 Australian Institute for Bioengineering and Nanotechnology, University of Queensland, St Lucia, QLD Australia

**Keywords:** Targeted gene repair, Cell delivery

## Abstract

Optimization of recombinant adeno-associated virus (rAAV) production is essential for effective gene therapy applications. However, multiple factors affect the rAAV productivity in mammalian cells, and often they interact with each other, making the optimization process highly challenging. In our previous work, we showed how coupling mixture design (MD) with face-centered central composite design (FCCD) was the most suitable design of experiments (DOE) approach for optimizing rAAV2 productivity and cell viability. In this study, we built on this method and demonstrate that combining MD with FCCD can be used to optimize the percentage of full capsids in rAAV2 upstream preparation. Additionally, we investigate the influence of the gene of interest (GOI) on the optimal conditions for viral particle production and packaging efficiency. By integrating MD and FCCD methodologies, we achieved an improvement of almost 100-fold in Log(Vp) in the case of egfp-expressing rAAV, and a 12-fold increase in bdnf-expressing full rAAV capsids, suggesting that this combined approach is a versatile and effective strategy for optimizing rAAV production processes. These findings emphasize the need for a comprehensive understanding of the factors influencing rAAV production to enhance the efficiency and efficacy of viral vector applications in gene therapy.

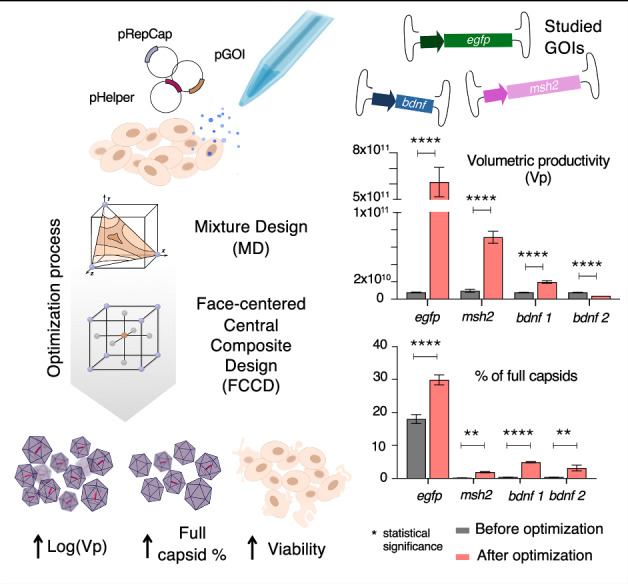

## Introduction

Recombinant adeno-associated viruses (rAAVs) have emerged as a leading vector system for gene therapies due to their capacity to deliver therapeutic genes with high efficiency, minimal immunogenicity, and prolonged expression in a variety of cell types. These vectors have shown promise in treating genetic disorders, cancers, and neurodegenerative diseases [1–3]. However, the production of rAAVs at a scale suitable for clinical and commercial applications remains a challenge, requiring optimized manufacturing processes that ensure high yield and quality [4].

In the rAAV production process, numerous factors influence the final yield and quality of the vectors. In biotechnological applications, the optimization of such complex processes can be effectively achieved using design of experiments (DOE). DOE is a structured methodology that explores the relationships between multiple factors and their effects on desired outcomes, facilitating the identification of optimal conditions. DOE has been used in various fields, including biotechnology, food manufacturing, agriculture, and material design. It allows researchers to study multiple variables and their interactions efficiently, reducing the number of experimental runs and providing robust statistical models for optimization [5–8]. Application of DOE to biological problems is challenging due to the high variability and complexity of biological systems. Traditionally, rAAV production optimization relied on the more time-consuming one-factor-at-a-time methods. DOE approaches have gained attention for their efficiency in optimizing rAAV production by systematically exploring different experimental parameters, mostly through Response Surface Methodology (RSM) approaches [9–11]. In our previous work [12], we suggested that the most suitable DOE approach for rAAV production optimization is a mixture design (MD) to select optimal plasmid concentrations, coupled with a face-centered central composite design (FCCD), to select optimal amount of DNA and transfection reagent.

When optimizing rAAV production, viability and volumetric productivity (Vp) are observed to be gene of interest (GOI)-dependent. Most studies in the field have employed green fluorescent protein (GFP) as GOI for the rAAVs [13]. GFP is an excellent model due to its easy quantification and non-toxicity to cells, making it a reliable marker for transfection and transduction efficiency and expression studies. However, in therapeutic contexts, using different GOIs might affect the metabolism of the producing cell, leading to unique impacts on rAAV production. Therefore, exploring the effects of various GOIs on rAAV production is imperative. In this study, we extend this investigation to include two additional genes of interest: MutS homolog 2 (*msh2)* and brain-derived neurotrophic factor *(bdnf)*. These GOIs have different lengths for the encapsidated constructs, ranging from 4.5 Kb in *msh2* to 2.5Kb for *bdnf*.

MSH2 protein is a key component of the DNA mismatch repair system, and mutations in *msh2* are associated with Lynch syndrome, a hereditary cancer predisposition condition, and other types of cancer [14–18]. BDNF plays a crucial role in neuronal survival, growth, and differentiation, and is linked to neurodegenerative diseases such as Alzheimer’s and Parkinson’s. rAAV-mediated delivery of BDNF has shown promise in neuroprotection in animal models for Huntington disease, after-stroke functional recovery, Prader-Willi syndrome and even major depression [19–22]. We have previously demonstrated that the GOI can significantly impact rAAV productivity [12]. In this study, we aim to investigate how different GOIs interact with the components of the triple transfection method to further elucidate the effects of GOIs on rAAV production. By choosing genes involved in diverse biological processes, we seek to provide a comprehensive analysis of the influence of various GOIs on rAAV production, while also demonstrating how MD coupled by an FCCD can serve as a versatile tool for investigating any GOI.

To evaluate the efficiency and quality of rAAV production, we focus on two critical responses: Vp and the percentage of full capsids (% full). Vp indicates the overall yield of the vector per unit volume, reflecting the efficiency of the production process in generating viral particles. This metric is crucial for understanding the productivity of the manufacturing process and ensuring that sufficient quantities of rAAVs are produced for therapeutic applications. % full, on the other hand, is a measure of the quality and efficacy of the produced rAAVs. In the process of rAAV production, not all generated viral capsids contain the full sequence of the GOI: a large percentage of the produced capsids are empty, while smaller percentages include a full or partial GOI sequence. Full capsids contain the functional therapeutic gene, while empty and partially full capsids do not. A high proportion of empty capsids can dilute the therapeutic effect and potentially trigger unwanted immune responses [23] and reduce transduction efficiency [24]. Therefore, optimizing the % full is essential for producing high-quality rAAVs that are both effective and safe for clinical use. This is a problem commonly tackled by downstream processing, where the upstream conditions for production are already fixed. Here, we show the importance of optimizing upstream conditions considering %full as a critical parameter.

## Materials and methods

### Cell culture

HEK293SF-3F6 cells from the National Research Council of Canada (NRCC) were cultivated in disposable polycarbonate 125-mL baffled shake flasks with vented caps (Corning® Life Sciences, USA) in 20 mL of HyCell TransFx-H media (Cytiva Life Sciences, USA), supplemented with 4 mM GlutaMAX™ (Gibco, Life Technologies, ThermoFisher, USA) and 0.1% Pluronic™ F-68 Non-ionic Surfactant (Gibco, Life Technologies). Cultures were maintained at 37 °C and 5% CO_2_ in a Hera Cell 150 incubator (ThermoFisher, USA) at an agitation of 130 rpm provided by Celltron (Infors HT, Switzerland).

Samples were collected for cell count and viability assessment at 48- and 72-h post-transfection (hpt) using NucleoCounter®-250 (Chemometec, Denmark) according to manufacturer’s instructions.

### Triple transient transfection in HEK293SF-3F6 cells

HEK293SF-3F6 cells were subjected to triple transfection using the following plasmids: pXR2 (pRepCap), pXX6-80 (pHelper) and PF1451 (pGOI-GFP)/ PF1452_BDNF (pGOI-BDNF)/ PF1451_MSH2 (pGOI-MSH2). pXR2 (National Gene Vector Biorepository, USA) contains the Rep and Cap genes from AAV2 and pXX6-80 (National Gene Vector Biorepository, USA) encloses the helper genes E2 and E4. PF1451 (Plasmid Factory, Germany) contains the gene-of-interest *egfp* flanked by AAV2 inverted terminal repeats (ITR), PF1452_BDNF (constructed by Azenta Life Sciences, United States) contains the gene-of-interest *bdnf* flanked by AAV2 ITR and PF1451_MSH2 (constructed by Azenta Life Sciences, United States) contains the gene-of-interest *msh2* flanked by AAV2 ITR. HEK293SF-3F6 were seeded at 1 × 10^6^ cells/mL the day prior to transfection to ensure that the cultures were at 2 × 10^6^ cells/mL when the transfection was performed. The transfection mix consisted in culture media, DNA and transfection reagent, all adding up to 5% of the total working volume. In this case, the working volume was 20 mL and the transfection mix 1 mL. Briefly, DNA was added to the corresponding volume of culture media, vortexed for 10 s and followed by the addition of the necessary amount of the transfection reagent FectoVIR®-AAV (FV) (Polyplus, France), all adding up to 150 µL. The transfection mix was vortexed 3 s for three times and incubated in accordance with the manufacturer’s instructions. The ratios between each plasmid and between the DNA and the transfection reagent were determined by the corresponding experimental design.

### Design of experiments (DOE) and models used

In this study, we analyzed the effect of viability, measured in percentage of live cells, on the production of rAAV, maximized the volumetric productivity (Vp), expressed in vg × mL^−1^ × day^−1^ and the full capsid percentage (full %) expressed in capsids × mL^–1^ × day^–1^. In order to fit the models, the Vp response was transformed to Log(Vp) as presented in **Equation 1** and described previously [12], using a mixture design (MD) coupled with a face-centered central composite design (FCCD).

1 $${Log}({V}_{P})={\log }_{10}\frac{{titer}\left(\frac{{vg}}{{mL}}\right)}{{time}({day})}$$

For each GOI, the ratios of pHelper, pGOI and pRepCap were optimized using MD and considering the mathematical constraint of all three components adding up to 100% of the mixture. To study the DNA:FV ratio, a two-factor FCCD was followed fixing the optimal plasmid ratio of the DNA mixture based on the results of the MD. Considering the nature of the triple transfection system, the designs were constrained to have a minimum quantity of each plasmid and FV to ensure successful production of rAAV.

The experimental design and statistical analysis were conducted using JMP 16 Pro (SAS Institute Inc., USA) for all designs.

#### Mixture design

The MD was defined as a mixture of the three required plasmids for the successful production of rAAV with a minimum of 10% and a maximum of 60% of each plasmid in the final mixture. Plasmids ratios and limits for all designs were measured in µg/mL. For the MD, a total amount of DNA of 2 µg/mL and FV ratio of 1:1 was used. Considering our constraints, a D-optimal design was selected as it minimizes the variance of the estimators. All factors were fitted to the quadratic formula presented below **(Equation 2)** where β corresponds to the expected response to the pure blend and *x*_i_ and *x*_j_ as the independent variables (pHelper, pRepCap and pGOI) where *x*_i_ = 1 and *x*_j_ = 0 when *j* ≠ *i*. *n* is the number of independent variables.

2 $$y={\sum }_{i=1}^{n}{\beta }_{i}{x}_{i}+{\sum }_{i=1}^{n}{\sum }_{j > i}^{n}{\beta }_{{ij}}{x}_{i}{x}_{j}$$

No blocking was required as all runs for every model could be quantified in one qPCR plate, avoiding batch effect [12]. Consequently, no random effect term was considered and the significance of the model was determined by an ANOVA test.

#### Response surface methodologies (RSM)

FCCD fits the experimental space to a second-order polynomial equation (**Equation 3)** where *y* is the response, either Log (Vp), full % or viability (%), β_0_ is the offset term, β_i_ is the linear coefficient, β_ii_ is the quadratic coefficient, β_ij_ is the interaction coefficient with *x*_i_ and *x*_j_ as the independent variable, ɛ is the noise observed in the response and *n* is the number of independent variables (total DNA amount and transfection reagent) .

3 $$y={\beta }_{0}+{\sum }_{i=1}^{n}{\beta }_{i}{x}_{i}+{\sum }_{i=1}^{n}{\beta }_{{ii}}{x}_{i}^{2}+{\sum }_{i=1}^{n}{\sum }_{j=i+1}^{n}{\beta }_{{ij}}{x}_{i}{x}_{j}+\varepsilon$$

Due to the high complexity of the systems, we used the desirability function (**Equation 4**) to identify the optimal values for the different responses [25]. For the desirability function, *T* is the target, *L* is the lower limit, *y* the response to be maximized and *r* is a number between 0 and 1 to show the relevancy of the response. To analyze more than one response simultaneously, such as viability, Log (V_P_) and full %, we merged both desirabilities in **Equation 5**, where *k* denotes the number of response variables (in this case, *k* = 3).

4 $$d=\left\{\begin{array}{cc}0\hfill &y < L\hfill \\ {\left(\frac{y-L}{T-L}\right)}^{r}&L\le y\le T\\ 1\hfill & y > T\hfill\end{array}\right.$$

5 $$D={d}_{1}^{1/k}{{\cdot }}{d}_{2}^{1/k}...{{{\cdot }}d}_{i}^{1/k}$$

All models were analyzed by ANOVA and their significance was assessed prior to experimental validation.

### rAAV quantification

Cell culture was harvested at 48 and 72 hpt and diluted 1:1 with lysis buffer (Tris-HCl 400 mM, Triton X-100 1% and MgCl_2_ 20 mM adjusted to pH 7.5). After 1 h incubation at 37 °C and agitation at 130 rpm, the lysate was centrifuged for 20 min at 4000 × *g* and 4 °C. The supernatants were stored at –70 °C for long-term storage.

On the quantification day, samples were thawed in a controlled manner at room temperature. Then, 5 µL of sample were mixed with 2 µL of 1 U/µL DNase I (ThermoFisher Scientific, USA) and 13 µL of 10X DNase I reaction buffer with MgCl_2_ (ThermoFisher Scientific). The mix was incubated for 16 h at 37 °C. To inhibit the activity of DNase I, 4 µL of 50 mM EDTA (ThermoFisher Scientific) were added to the mix followed by a 30 min incubation at 70 °C. Lastly, 5 µL of Proteinase K (ThermoFisher Scientific) were added to the sample and incubated for 2 h at 55 °C. The Proteinase K was inactivated for 15 min at 95 °C. 2 µL of the freshly treated sample were mixed with 0.5 µL of 10 µM forward primer (5ʹ-ACGTCAATGGGTGGAGTATTT-3ʹ) and reverse primer (5ʹ- AGGTCATGTACTGGGCATAAT-3ʹ) binding to the e*gfp* sequence with 5 µL of Brilliant III Ultra-Fast SYBR Green QPCR Master Mix (Agilent Technologies, USA) and 0.15 µL of 2 µM reference dye. Respectively, forward primer (5ʹ- GAACCTCCACTCCTGTTTCTG -3ʹ) and reverse primer (5ʹ- ACCGCACTCTCATGCTCATATT -3ʹ) were used for binding to the *bdnf* sequence and forward primer (5ʹ- GTTCGGCTACTACTTCAGAGTG -3ʹ) and reverse primer (5ʹ- AACTTCACGCCGTTCTTCT -3ʹ) for binding to the *msh2* sequence.

Amplification was executed in QuantStudio 5 Real-Time PCR System (ThermoFisher Scientific) with the following conditions: 50 °C for 2 min, 95 °C for 10 min; 40×: 95 °C for 15 s, 60 °C for 1 min, 95 °C for 15 s, 60 °C for 1 min and 95 °C for 1 s.

In order to ensure the reliability of the generated data, two controls were added: saturate lysate control (SLC) and transfected lysate control (TLC). The SLC was prepared from a non-transfected cell culture which underwent the lysis protocol in parallel with the transfected samples. Then 5 µL of cell lysate were mixed with 40 ng of pGOI plasmid, 9 µL of 10X DNase I reaction buffer with MgCl_2_ and 2 µL of 1 U/µL DNase I. SLC control aimed to verify that the DNase step was successful. For the generation of the TLC, a single plasmid transfection was performed with pGOI following the triple transfection, lysis and the quantification protocol. The TLC aimed to guarantee that the effect of DNase I remains independent of whether the plasmid has been transfected or spiked afterwards. Plates run in QuantStudio 5 Real-Time PCR System were analyzed via ThermoCloud (ThermoFisher Scientific).

Capsid quantification was conducted using an AAV2 titration ELISA kit (PROGEN, Heidelberg, Germany) following manufacturer’s instruction in order to calculate the percentage of full capsids (% full) in the vector preparation.

### DNA sequence analysis

DNA Secondary Structure Prediction Tool by VectorBuilder (https://en.vectorbuilder.com/tool/dna-secondary-structure.html) was used to analyse the potential secondary structure of the rAAV genome sequences of the three rAAV constructs. GC Content Calculator by VectorBuilder (https://en.vectorbuilder.com/tool/gc-content-calculator.html) was used to measure the GC percentage and change in Gibbs free energy (∆G) of the rAAV genome sequence of the three rAAV constructs.

## Results

### The rAAV optimization process varies based on the gene of interest

**Fig. 1:**
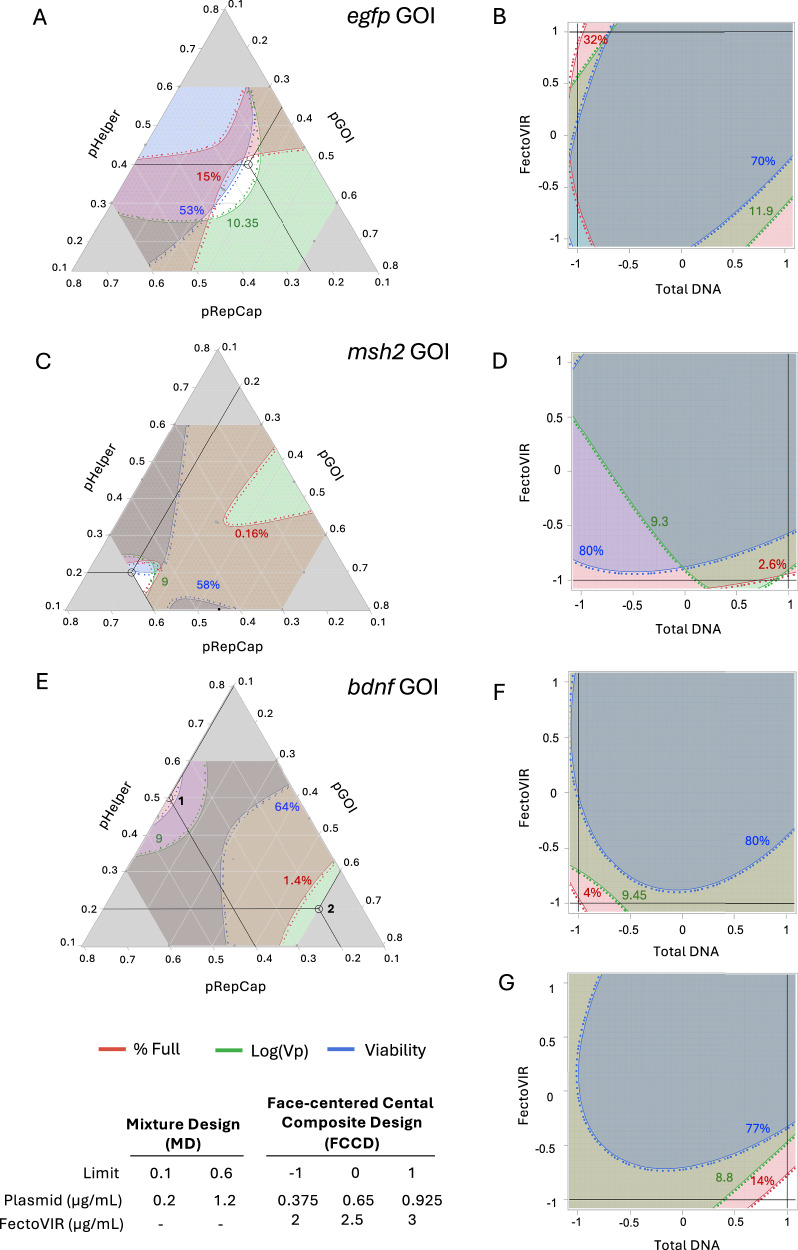
Graphical representation of experimental spaces, showing how % full (red), Log(Vp) (green) and viability (blue) change at different ratios. Ratios are calculated in mass (µg/mL). The shadowed areas represent the experimental space that is below the selected threshold for each of the three responses; green for Log(Vp), blue for viability and red for % full. The dotted lines represent the selected thresholds for each response. The threshold values are indicated in their corresponding colours. The white areas represent the experimental space where the three responses exceed the thresholds, meaning that they are optimal areas. The threshold values were determined by the desirability function (Eq. 5) to have equal weight of the three responses. The table on the bottom left corner shows the limits in coded notation (from 0 to 1) of the MD and FCCD models for each variable. **A** Mixture profiler in MD plasmid optimization for *egfp*-expressing rAAV for the responses of % full, Log(Vp) and viability. Chosen point is 0.4:0.25:0.35 (pHelper:pRepCap:pGOI). **B** FCCD contour plot for total DNA and FV optimization for *egfp*-expressing rAAV. Chosen point is -1,1 (DNA,FV). **C** Mixture profiler in MD plasmid optimization for *msh2*-expressing rAAV for the responses of % full, Log(Vp) and viability. Chosen point is 0.2:0.6:0.2. **D** FCCD contour plot for total DNA and FV optimization for *msh2*-expressing rAAV. Chosen point is 1,-1 (DNA,FV). **E** Mixture profiler in MD plasmid optimization for *bdnf*-expressing rAAV for the responses of % full, Log(Vp) and viability. 2 points were chosen. Point 1 aims to optimize Log(Vp) and is 0.5:0.4:0.1 (pHelper:pRepCap:pGOI). Point 2 aims to optimize % full and is 0.2:0.2:0.6 (pHelper:pRepCap:pGOI). **F** FCCD contour plot for total DNA and FV optimization for *bdnf*-expressing rAAV (case 1- Log(Vp) focused). Chosen point is -1,-1 (DNA,FV). (G) FCCD contour plot for total DNA and FV optimization for *bdnf*-expressing rAAV (case 2- % full focused). Chosen point is 1,-1 (DNA,FV).

For a given GOI, the optimal Log(Vp) and optimal % full were not always achieved with the same plasmid ratios (Fig. 1), varying across the experimental space. This discrepancy introduces challenges in the optimization process, as focusing only on productivity for optimization may lead to suboptimal full-to-empty ratio in the selected optimum.

When using *egfp* as the GOI, MD revealed that Log(Vp) is higher with high pHelper and pRepCap levels and low pGOI levels, while cell viability decreased in these conditions (Fig. 1A). In contrast, optimal values for the % full response were found in regions where neither Log(Vp) nor viability were maximized simultaneously (Fig. 1A). Due to the significant reduction in viability where Log(Vp) and % full responses were the highest and, considering the potential impact of low viability on product quality, we selected a plasmid ratio of 0.4:0.25:0.35 (pHelper:pRepCap:pGOI) as the optimal condition considering the three different responses. This was followed by further optimization of the total DNA and transfection reagent quantities. In the subsequent FCCD, it was found that viability did not limit the optimal values. Log(Vp) was higher when FV levels were low and DNA levels were high, or when FV levels were high, and DNA levels were low. However, % full was found to be higher when FV levels were high, and DNA levels were low. Thus, -1,1 (DNA,FV) was chosen as the optimal point. (Fig. 1B).

In the case of *msh2* as a GOI, a different pattern emerged. The optimal conditions for all three responses (Log(Vp), % full, and viability) were found when pHelper and pGOI levels were lower and pRepCap levels were higher, with an optimal plasmid ratio of 0.2:0.6:0.2 (Fig. 1C). In the FCCD, lower concentrations of the transfection reagent FV significantly improved the % full response, with DNA levels having a minimal impact. However, for high FV levels, higher DNA concentrations reduced the % full response. Log(Vp) increased as FV concentrations decreased, with maximum values observed when both FV and DNA were at their lower limits. Despite this, high Log(Vp) was still supported when total DNA was high and FV was low (Fig. 1D). Considering the effect on viability, which peaked when FV was low and DNA was high, the 1,-1 (DNA, FV) conditions were chosen as optimal (Fig. 1D).

The optimization landscape for *bdnf* was more complex. A saddle point was observed for viability, but it did not severely limit the selection of optimal values. However, the other two responses, Log(Vp) and % full, moved in opposite directions across the experimental space (Fig. 1E). Log(Vp) was higher at high pHelper and pRepCap levels and decreased as pGOI increased. In contrast, the % full response improved with higher pGOI and lower pHelper and pRepCap levels. To accommodate these conflicting trends, two distinct FCCD designs were implemented for each of the two optimal ratios. One design focused on optimizing Log(Vp) (Fig. 1F), while the other targeted optimization of the full-to-empty capsid ratio (Fig. 1G). All responses were measured in both designs. In both cases, low FV levels optimized all responses. However, in the Log(Vp)-optimized design, low DNA levels yielded higher values across all responses. Therefore, -1,-1 (DNA, FV) was chosen as the optimal point to optimize Vp (Fig. 1F), whereas in the % full optimized design, higher DNA levels led to the best outcomes leading to 1,-1 (DNA, FV) being chosen as optimal for % full (Fig. 1G).

These findings demonstrate that the optimization process is highly dependent on the specific GOI, as each one influences the plasmid ratios and conditions required to achieve optimal responses. Furthermore, the optimal values for one response do not necessarily coincide with those for another response, highlighting the need for tailored optimization strategies for each GOI.

### Optimizing viral particle production reveals GOI-specific variability in percentage of full rAAV capsids

**Fig. 2:**
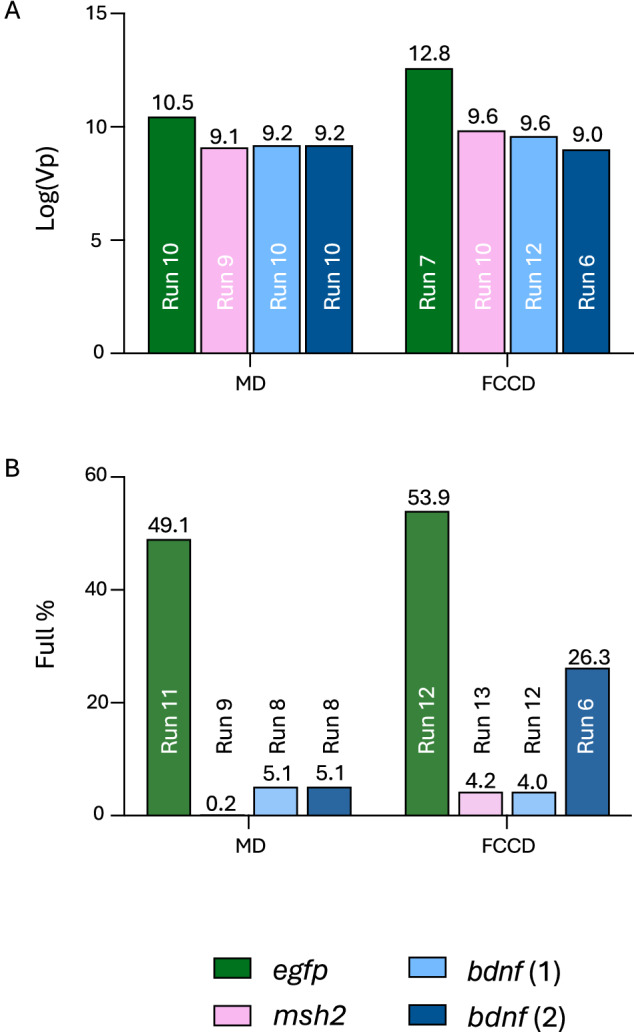
Optimal values achieved within the experimental runs. **A** Optimal values achieved for Log(Vp) for *efgp/msh2/bdnf*-expressing rAAV for MD and FCCD. **B** Optimal values achieved for % full for *efgp/msh2/bdnf*-expressing rAAV for MD and FCCD.

**Fig. 3:**
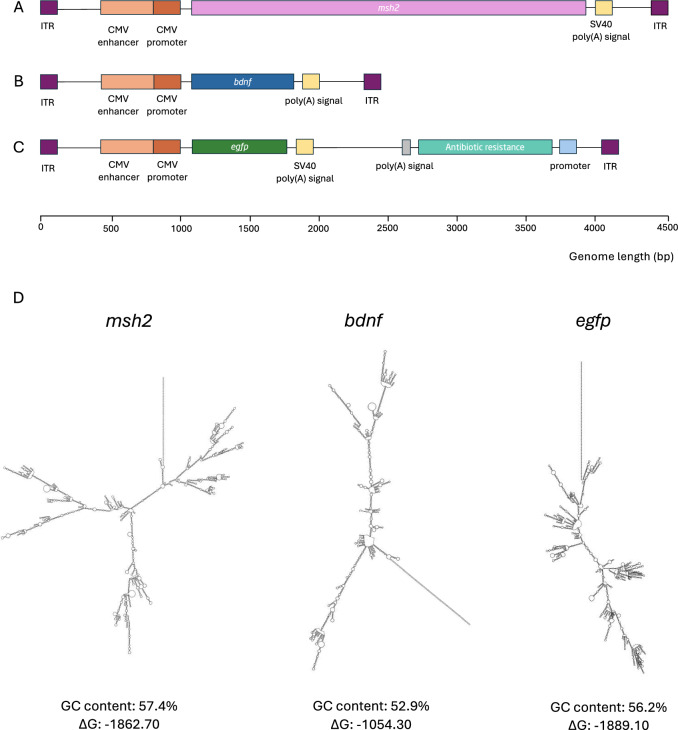
Structure of rAAV constructs. **A** *msh2*-expressing rAAV construct. **B** *bdnf*-expressing rAAV construct. **C** *egfp*-expressing rAAV construct. **D** Secondary structure of studied rAAV constructs and features of GC content and ΔG. Left: secondary structure of *msh2*-expressing vector genome, center: secondary structure of *bdnf*-expressing vector genome, right: secondary structure of *egfp*-expressing vector genome.

The optimal values achieved in the experimental runs for Log(Vp) were relatively consistent across the three different GOIs. In terms of Log(Vp), the optimal values in the MD ranged from 9.2 for *bdnf* to 10.46 for *egfp*, while after optimizing FCCD for Log(Vp), the range improved from 9.6 for *bdnf* to 12.6 for *egfp* (Fig. 2A). This suggested that viral particle production, as measured by Log(Vp), is less sensitive to the specific GOI, observing a more uniform response in this parameter across the experimental space. However, the full-to-empty ratio showed substantial differences depending on the GOI. In the MD, optimal values varied significantly, from as low as 0.18% for *msh2* to 49% for *egfp*, while after the FCCD, the range extended from 4.2% for *bdnf* to 54% for *egfp* (Fig. 2B). These larger deviations highlight that the formation of full viral particles is highly dependent on the GOI. The differences observed, particularly in the % full response, emphasize the need for GOI-specific optimization strategies, suggesting that the efficiency of packaging full viral genomes is more variable, likely due to the distinct biological characteristics of each GOI. To get a further understanding of factors potentially affecting rAAV genome encapsidation that are distinct for each GOI, we performed an in silico analysis of the DNA sequence flanked by the ITRs in each rAAV construct. This analysis included GC sequence content, predictions of ∆G, the rAAV genome secondary structure and construct length. While GC content, ∆G and construct length did not show any correlation to rAAV genome encapsidation efficiency, our analysis revealed that wider secondary structures (*msh2*, *bdnf*) are correlated with lower % full values (Fig. 3D), suggesting that rAAV genome secondary structure might affect the formation of full viral particles.

### FCCD can improve Log(Vp) and % full for three different GOIs

In our previous work, we highlighted the ability of FCCD to improve Log(Vp) and viability of cell producing *egfp*-expressing rAAV. Here, we focused on the response of Log(Vp) and % full and showed that FCCD can be used to improve Log(Vp) and % full responses not only for *egfp*-expressing rAAV, but for two more GOIs, after optimization with a MD. The highest Log(Vp) values obtained for *egfp*-expressing rAAV was 12.6 after FCCD, compared to 10.46 after MD, while for *msh2*-expressing rAAV, FCCD runs (Supplementary Table S1) reached 9.85, compared to 9.1 after MD. When optimizing for Log(Vp) for *bdnf*-expressing rAAV, the highest Log(Vp) values after FCCD (Supplementary Table S3) reached 9.6, while after MD the highest value was 9.2 (Fig. 2A).

Similarly, we obtained 54% full *egfp*-expressing rAAVs after FCCD (Supplementary Table S2), 5% higher than after MD. The highest % full value for *msh2*-expressing rAAVs during FCCD runs was 4.22%, a 23-fold increase compared to the optimal values obtained with MD. When optimizing for high % full for *bdnf*-expressing rAAVs, the highest achieved value with FCCD was 26.2%, compared to 5.13% obtained with MD, a 5-fold improvement (Fig. 2B).

### Model validations and process improvement

**Fig. 4:**
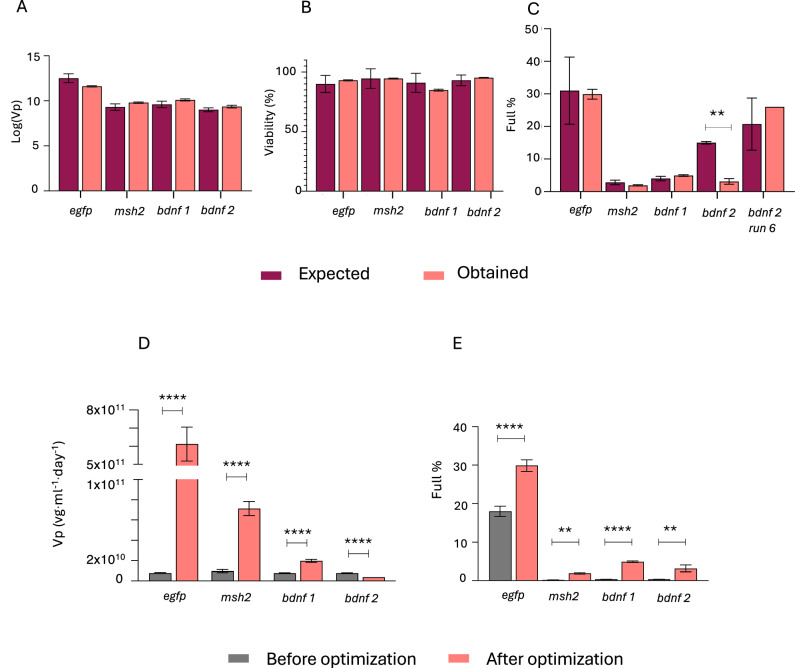
Experimental validations. Predicted and actual experimental values for all validated conditions for all models regarding (**A**) log(Vp), (**B**) viability and (**C**) % full. Significant differences (*p* < 0.05, unpaired *t* test) were found only between the expected and obtained values for *bdnf*-expressing rAAV, in the case of % full optimizing FCCD model, validating most models. Vp and full % improvement across all tested constructs are presented in (**D**) and (**E**), respectively. In most cases significant improvement (*p* < 0.05, unpaired *t* test) was observed. The data are presented as the mean ± SD (*p  <  0.05, **p  <  0.01, ***p  <  0.001). *bdnf 1* represents the optimal value to maximize Vp and *bdnf 2* represents the optimal value to maximize % full.

To validate the predicted responses of the optimal values from each DOE model, validation runs were performed, with the results demonstrating that the predicted outcomes closely align with the observed responses for most genes and responses, with no significant differences, supporting the robustness of the MD + FCCD approach. For Log(Vp), the expected and obtained values were highly consistent for all genes, with only minimal, non-significant deviations observed, indicating strong predictive accuracy for this parameter (Fig. 4A). In terms of viability, all constructs maintained high cell viability, with no significant differences between the expected and obtained values (Fig. 4B). For the % full response, the predictions were also closely aligned with the observed values in most cases. However, for *bdnf* 2, the predicted value deviated significantly from the obtained. The results indicate that the optimal conditions do not achieve the expected response for % full (Fig. 4C). Interestingly, the tested condition of 1,-1 (DNA,FV) in *bdnf 2* FCCD - which corresponded to run 6 - showed a more accurate outcome for % full, with predicted values closely aligning with the obtained response. The other two responses were also predicted accurately from the model (Supplementary Fig. 1), only Log(Vp) was lower than the predicted optimal presented in Fig. 4B. Therefore, these conditions should be utilized for optimizing only % full response, in case this is the only response of interest, as it yields better performance compared to the initially predicted optimal condition.

Lastly, we compared our improved conditions with conditions before optimization (plasmid ratio 1:1:1 for pHelper:pRepCap:pGOI and 1:1 DNA:FV), Our results underscored the versatility of the MD + FCCD approach for achieving overall optimization from non-optimized to fully optimized production. Specifically, the final optimization using MD + FCCD led to substantial increases in both Vp and % full particle production compared to non-optimized transfection conditions. For *egfp*-expressing rAAVs, the Vp improved by almost 100-fold (Fig. 4D), and the full % increased by 1.6-fold (Fig. 4E). The case of *msh2*-expressing rAAVs showed significant improvements, with a tenfold increase in Vp (Fig. 4D), accompanied by a tenfold increase in full % (Fig. 4E), demonstrating effective optimization of both particle production and packaging efficiency. Notably, in the case of *bdnf* 1, which focuses on optimization of Vp, there was a significant threefold improvement in the Vp (Fig. 4D), together with a 12-fold improvement in % full (Fig. 4E). In the case 2 of *bdnf*, where the optimization was focused on increasing % full particle production, a decrease in Vp was observed (Fig. 4D), which was anticipated given the emphasis on packaging efficiency, together with a significant 7.8-fold improvement for % full response (Fig. 4E). This case underscores the importance of integrating both Vp and % full as parallel objectives during the optimization process, as reduction in Vp optimization could be observed when prioritizing only full %, compromising the total yield and efficacy of the final product.

## Discussion

### Design space flexibility across mass and molar plasmid ratios

Plasmid ratios were defined based on mass (µg/mL). While this results in different copy numbers depending on plasmid size, the design space is sufficiently broad to capture equivalent molar ratios. For example, the 0.4:0.35:0.25 µg/mL ratio for *egfp* corresponds to 1.92 × 10^10^, 4.26 × 10^10^, and 2.89 × 10^10^ copies/mL, respectively. Applying the same copy numbers for a different GOI, such as *bdnf*, results in a mass ratio of approximately 0.3:0.35:0.2 µg/mL, still within the studied range. Thus, the design allows identification of optimal conditions regardless of whether mass or molar ratios are used.

### Tailored optimization of Log(Vp) and % full is needed in rAAV preparation

Our results indicate that the optimal conditions for Log(Vp), measuring overall viral particle productivity, and % full, measuring the proportion of fully packaged viral particles, can vary significantly depending on GOI. This divergence in optimal values highlights an important consideration for viral vector production and optimization. If optimization targets focus solely on maximizing Log(Vp), there is a risk of producing large quantities of viral particles that are predominantly empty. Although Log(Vp) reflects total particle output, it does not account for the critical factor of genome packaging, and using Log(Vp) as the main response to optimize could be misleading in terms of actual product quality. Without considering % full, the final yield of functional viral particles after purification could be considerably lower than anticipated. Therefore, it is essential to take both Log(Vp) and % full responses into account when optimizing upstream production, as focusing exclusively on overall productivity may result in a high volume of non-functional particles and a reduced effective yield.

In some cases, both responses may align, simplifying the optimization process. However, when the optimal values for Log(Vp) and % full differ, it is essential to carefully balance the two responses based on the specific goals of production. This underscores the need for a more holistic optimization approach, ensuring that both total particle production and the formation of full viral particles are considered to achieve the best outcome in terms of both quantity and quality of the final viral vector preparation.

### GOI-dependent cellular functions and structure of vector genome might affect rAAV production and packaging

The effects that each GOI exerts on the producer cells could also affect the production and packaging of AAV genome into the capsid and the % full response. The genes selected for this study, *egfp, msh2, and bdnf*, have diverse gene functions that may differentially influence cellular pathways and processes, potentially impacting the efficiency of viral genome replication, particle assembly and packaging.

EGFP is a small, non-toxic fluorescent protein, widely used in biological studies due to its minimal impact on cellular physiology [13]. This may explain why EGFP consistently leads to higher % full and Log(Vp) values, as the producer cells likely experience minimal disruption of their normal functions during transfection. In contrast, *msh2*, a DNA mismatch repair protein, plays a critical role in maintaining genomic integrity. Overexpression of *msh2* gene might interfere with the DNA repair machinery and reduce the cell’s capacity to support the efficient production of fully packaged viral particles, as the host cell machinery may be diverted to repairing or processing mispaired bases, especially in cells already undergoing the stress of transfection [26]. Additionally, overexpression of *msh2* gene might induce cell apoptosis in human cell lines under the control of CMV promoter, increasing the system’s complexity and potentially further limiting viral production and particle packaging efficiency, contributing to the lower % full values observed [27]. BDNF plays a well-established role in neurons, but its influence extends to other cell types, including immune and muscle cells, where it regulates essential metabolic functions. Overexpression of the *bdnf* gene can affect lipid metabolism, leading to a reduction in energy intake and affect overall cellular energy homeostasis. This impact on cellular metabolism may result in alterations to the resources available for processes like AAV genome replication, capsid assembly and packaging, which require substantial energy and resources from the cell [28]. Overall, it is expected that GOIs can affect the production of functional viral particles through their biological functions and interactions with host cell metabolism.

While the length of the AAV genome between the ITRs might influence encapsidation efficiency, our results suggest that genome size alone does not fully account for the observed differences in packaging yields. For instance, although the constructs containing *efgp* and *msh2* are of comparable length, the resulting % full differ importantly. This indicates that additional factors beyond size likely contribute to encapsidation efficiency. One possible explanation is the influence of sequence-specific features such as secondary structure, which could affect genome folding, stability, or recognition during packaging.

The AAV ITRs adopt a specific secondary structure and constitute the origin of replication in the AAV genome, but also contain sequences that are critical for genome translocation into the AAV capsid [4]. However, different GOI sequences could have a distinct secondary structure, changing the accessibility of the packaging signals contained in the ITRs. The vector genome of both *msh2*- and *bdnf*- expressing rAAVs is predicted to have a wider and more complex secondary structure than the predicted secondary structure of the *egfp*-expressing vector genome (Fig. 3D–F). Potential steric hindrance could affect the accessibility of packaging signals within the ITRs and challenge the translocation of the rAAV genome into the viral capsids. *egfp*-expressing rAAV preparations reach the maximum % full and this construct is predicted to have the most linear secondary structure compared to the other two. Between *bdnf*- and *msh2*- expressing constructs, the first one is predicted to have more narrow structure and reaching higher % full values, suggesting that rAAV genome secondary structures could potentially affect rAAV packaging. Xie et al. showed that specific genomic secondary structures increase the events of genome truncation during translocation into the rAAV capsid, highlighting the importance of secondary genome structure in rAAV genome encapsidation [29]. However, more research would be needed to further clarify the role of it in the encapsidation efficiency.

### Coupling MD with FCCD is a versatile strategy for upstream optimization of multiple responses

In our current study, we extended the application of the MD + FCCD strategy to the optimization of % full rAAV particles, demonstrating its effectiveness not only for improving Log(Vp) but also for further enhancing rAAV packaging efficiency. By coupling FCCD with MD, we achieved a comprehensive decoupling of biological factors influencing plasmid interactions, enabling precise fine-tuning of full viral particle production beyond the initially improved values achieved with MD. This integration highlighted the critical importance of total DNA and transfection reagent amounts, as these parameters had a substantial impact on particle production and packaging efficiency. Across all tested GOIs, coupling further FCCD optimization led to improvements in % full, with a maximum 23-fold increase observed for *msh2*-expressing rAAVs and a 5-fold improvement for *bdnf*-expressing rAAVs compared to the optimal values achieved with MD alone. These results underscore that while MD effectively determines optimal plasmid ratios to address biological factors, incorporating FCCD allows for a deeper analysis of additional experimental conditions.

Optimizing the responses of Log(Vp) and %full during rAAV production is critical, as it ensures efficient and effective processing throughout the system. Most research studies so far aim to address low percentage of full capsids through improvement in downstream processing [30–35]. This approach can be both costly and time-consuming, requiring extensive adjustments to compensate for upstream inefficiencies. Our study further highlights the importance of upstream optimization and the significance of upstream conditions in full rAAV production. Here we show that upstream optimization can tackle this issue and significantly improve the % full response. Optimizing upstream processes not only enhances the initial response rates but also simplifies downstream workflows, reducing the need for complex corrections and ensuring a more streamlined and cost-effective production process.

### Scalability and process transfer considerations

The primary goal of optimizing plasmid ratios at lab scale is to establish a solid foundation for transfer to bioreactor-scale production. While we expect the upscaled system to exhibit similar trends in productivity, process performance at larger scale can be influenced by additional factors such as mixing, mass transfer and pH control. Therefore, further fine-tuning may be necessary and should be guided by experimental validation. Nonetheless, the optimized conditions identified here provide a robust starting point for efficient and scalable rAAV manufacturing.

## Supplementary information

Supplementary Table S1

Supplementary Table S2

Supplementary Table S3

Predicted and actual experimental values for all validated conditions

## Data Availability

Raw data from viability, titer and full-to-empty ratio measurements for each run can be found in the supplementary tables. No datasets were deposited in a public repository
