# Supplementary material for: Mixture design as a tool for improving full-to-empty particle ratios across various GOIs in rAAV production
Source: Gene Ther. 2025 Jun 20;33(1):48–56. doi: 10.1038/s41434-025-00546-5 (PMC12932106; doi:10.1038/s41434-025-00546-5)
Supplement: Supplementary file 1 — Supplementary Table S1 [file 41434_2025_546_MOESM1_ESM.pdf]

Supplementary Table S1: Matrices and responses for msh2 DoE designs. The limits for each factor are represented with the coded notation from 0 to 1. The three analyzed responses are shown on the right, with the resulting values for each run. The Pattern colum for the FCCD design shows the limit combination for the run.

msh2 Mixture Design

Run	pHelper	pRepCap	pGOI	Log(Vp)	Viability	Full capsids (%)
1	0.1	0.4465975	0.4534025	8.55	57.2	0.08
2	0.333435	0.333435	0.3331295	8.92	55.8	0.12
3	0.6	0.1	0.3	8.67	65.3	0.11
4	0.1	0.6	0.3	8.92	55.7	0.15
5	0.4439035	0.1	0.4560965	8.82	64.1	0.19
6	0.3	0.6	0.1	9.03	57.9	0.14
7	0.45	0.45	0.1	8.57	58.3	0.05
8	0.2	0.2	0.6	7.59	58.6	0.01
9	0.3355615	0.3355615	0.328877	9.13	61.3	0.22
10	0.6	0.3	0.1	8.09	53.4	0.02
11	0.1	0.3	0.6	8.42	61.6	0.06
12	0.3	0.1	0.6	8.75	62	0.16

msh2 FCCD

Run	Pattern	Total DNA	FectoVIR	Log(Vp)	Viability	Full capsids (%)
1	++	1	1	8.32	54.3	0.04
2	0A	0	1	8.65	60.5	0.11
3	0	0	0	8.80	61.0	0.14
4	0	0	0	8.85	59.8	0.16
5	A0	1	0	9.03	65.2	0.20
6	+−	1	-1	9.23	94.0	2.57
7	−+	-1	1	8.77	77.4	0.44
8	0	0	0	8.98	59.9	0.19
9	0	0	0	8.88	60.2	0.13
10	a0	-1	0	9.85	78.0	3.22
11	0	0	0	8.95	62.0	0.19
12	−−	-1	-1	9.46	78.1	0.98
13	0a	0	-1	9.60	90.8	4.23
