# Supplementary material for: Mixture design as a tool for improving full-to-empty particle ratios across various GOIs in rAAV production
Source: Gene Ther. 2025 Jun 20;33(1):48–56. doi: 10.1038/s41434-025-00546-5 (PMC12932106; doi:10.1038/s41434-025-00546-5)
Supplement: Supplementary file 4 — Predicted and actual experimental values for all validated conditions [file 41434_2025_546_MOESM4_ESM.pdf]

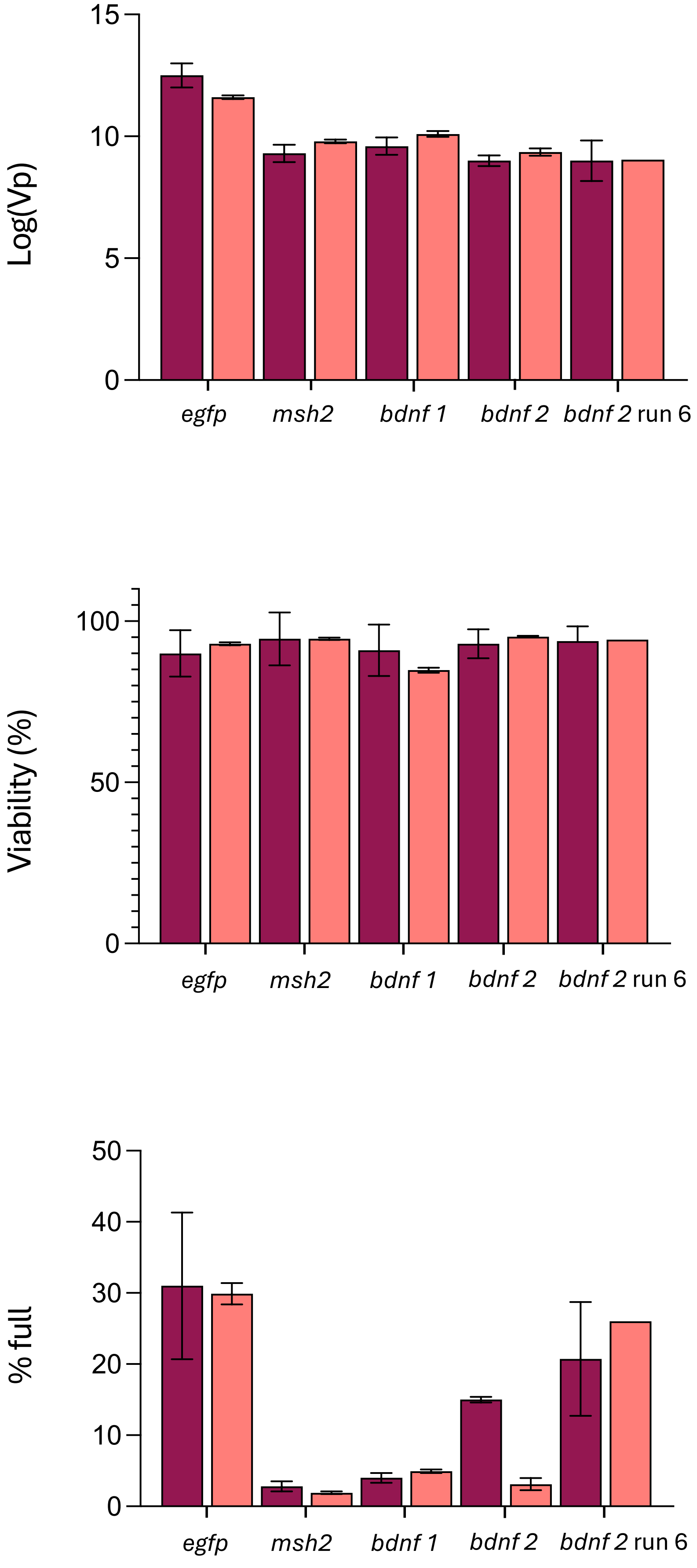

Supplementary Fig. 1: Predicted and actual experimental values for all validated conditions for all models regarding (A) log(Vp), (B) viability and (C) % full. Significant differences ($p<0.05$, unpaired t-test) were found only between the expected and obtained values for bdnf-expressing rAAV, in the case of % full optimizing FCCD model, validating most models. Data from run 6 in the case of *bdnf* 2 are presented in all responses.
